# Digestive and appendicular soft-parts, with behavioural implications, in a large Ordovician trilobite from the Fezouata Lagerstätte, Morocco

**DOI:** 10.1038/srep39728

**Published:** 2017-01-10

**Authors:** Juan C. Gutiérrez-Marco, Diego C. García-Bellido, Isabel Rábano, Artur A. Sá

**Affiliations:** 1 Instituto de Geociencias (CSIC, UCM) and Departamento de Paleontología, Facultad de Ciencias Geológicas, José Antonio Novais 12, 28040 Madrid, Spain; 2 School of Biological Sciences, University of Adelaide, South Australia 5005, Australia; 3 South Australian Museum, South Australia, 5000, Australia; 4 Museo Geominero, IGME, Ríos Rosas 23, 28003 Madrid, Spain; 5 Departamento de Geologia, Universidade de Trás-os-Montes e Alto Douro, Quinta de Prados 5001-801 Vila Real, Portugal; 6 Centro de Geociências, Universidade de Coimbra, Polo II, 3030-790 Coimbra, Portugal

## Abstract

Trilobites were one of the most successful groups of marine arthropods during the Palaeozoic era, yet their soft-part anatomy is only known from a few exceptionally-preserved specimens found in a handful of localities from the Cambrian to the Devonian. This is because, even if the sclerotized appendages were not destroyed during early taphonomic stages, they are often overprinted by the three-dimensional, mineralised exoskeleton. Inferences about the ventral anatomy and behavioural activities of trilobites can also be derived from the ichnological record, which suggests that most *Cruziana* and *Rusophycus* trace fossils were possibly produced by the actions of trilobites. Three specimens of the asaphid trilobite *Megistaspis (Ekeraspis) hammondi*, have been discovered in the Lower Ordovician Fezouata Konservat-Lagerstätte of southern Morocco, preserving appendages and digestive tract. The digestive structures include a crop with digestive caeca, while the appendages display exopodal setae and slight heteropody (cephalic endopods larger and more spinose than thoracic and pygidial ones). The combination of these digestive structures and the heteropody has never been described together among trilobites, and the latter could assist in the understanding of the production of certain comb-like traces of the *Cruziana rugosa* group, which are extraordinarily abundant on the shallow marine shelves around Gondwana.

The Moroccan Fezouata Biota is the most important Ordovician Burgess Shale-type Lagerstätte known to date, having produced a very diverse marine assemblage, which includes not only articulated mineralized taxa, but also soft-bodied or lightly sclerotized organisms (e.g, annelids, priapulids, lobopodians)^1^,^2^. Preservation usually involves pyritization and subsequent weathering to iron oxides, thus the reddish colours of the fossils. The assemblage has been dated as late Tremadocian (~478 Ma), from relatively shallow waters, at or just above the storm-wave base^3^. Among the fossil arthropods, preserved appendages are frequent in the following groups: anomalocaridids, marrellomorphs, xyphosurans, aglaspidids, etc.^2^,^4^. Additionally, fossilized digestive tracts have been described in machaeridian annelids^5^.

Trilobites are common constituents of the Fezouata Biota, found as complete carcasses as well as putative articulated exuviae. However, soft-body preservation is extremely rare, with only antennae and some distal appendage remains recognized in *Bavarilla*^3^ and the nileid *Symphysurus*, where a single specimen of the latter exceptionally showed “preserved antenna, walking legs and midgut glands”^1^. Here we present the first occurrence of the preserved gut with associated digestive structures, plus a complete set of endopods and exopods in specimens of the asaphid trilobite *Megistaspis (Ekeraspis) hammondi*. They come from site “F-4”^6^, which is located about 7 km north of the Bou Izargane village and 19 km north of the city of Zagora, central Anti-Atlas of Morocco (Supplementary Fig. 1). At this locality, large trenches have been excavated in search of fossils, for scientific and commercial purposes, which were deposited during the late Tremadocian *Araneograptus murrayi* graptolite Biozone. The studied trilobites were collected by the Ben Moula family (Taychout village, near Alnif-Tinghir, Morocco), later bought by a professional fossil dealer and offered to the *Museo Geominero* of the Spanish Geological Survey, in Madrid (catalogue numbers MGM-6755X to MGM-6757X). Restoration of fossil material from Morocco is common, as it is often intended for amateur collectors. However, extreme care has been taken by the authors in authenticating the material studied here. Only specimens collected by reputable sources have been considered, as is the case of the Ben Moula family, who have provided the best Fezouata material^1^,^2^,^3^,^5^. These specimens have later been re-checked for consistency in lithology, colouration and slab thickness to ensure their authenticity and integrity. The areas that have been retouched in any form in the specimen of Fig. 1a have a lighter shade and outlined in red, and this restoration has not affected either the digestive structures or the diagnostic features. The details of the type of restoration in the specimen are indicated in Supplementary Fig. 2a, with the trilobite having ~10% of extensively reconstructed parts, ~12% of slightly reconstructed parts, ~18% of polished surfaces of the internal mould and ~5% of pieces transferred from the external mould (shown in Supplementary Fig. 2b).

*Megistaspis (Ekeraspis) hammondi*^7^ (=*M. (E.*) cf. *filacovi*^8^,^9^) is a medium-large asaphid trilobite, with eight thoracic segments and a pygidium with a long caudal spine (Fig. 1a). It differs from the remaining species of the subgenus in the more anterior position of the eyes.

The original description of the species needs revision, because the diagnostic characters are barely recognizable on the holotype^7^, and the purported differences of the subspecies *M. (E.) hammondi forteyi*^7^ could be due to preparation by the fossil dealers (Supplementary Fig. 3a,b). Thus, the latter could be synonimized with the *forma typica* of *M. (E.) hammondi*.

## Results

### Digestive system

**Figure 1:**
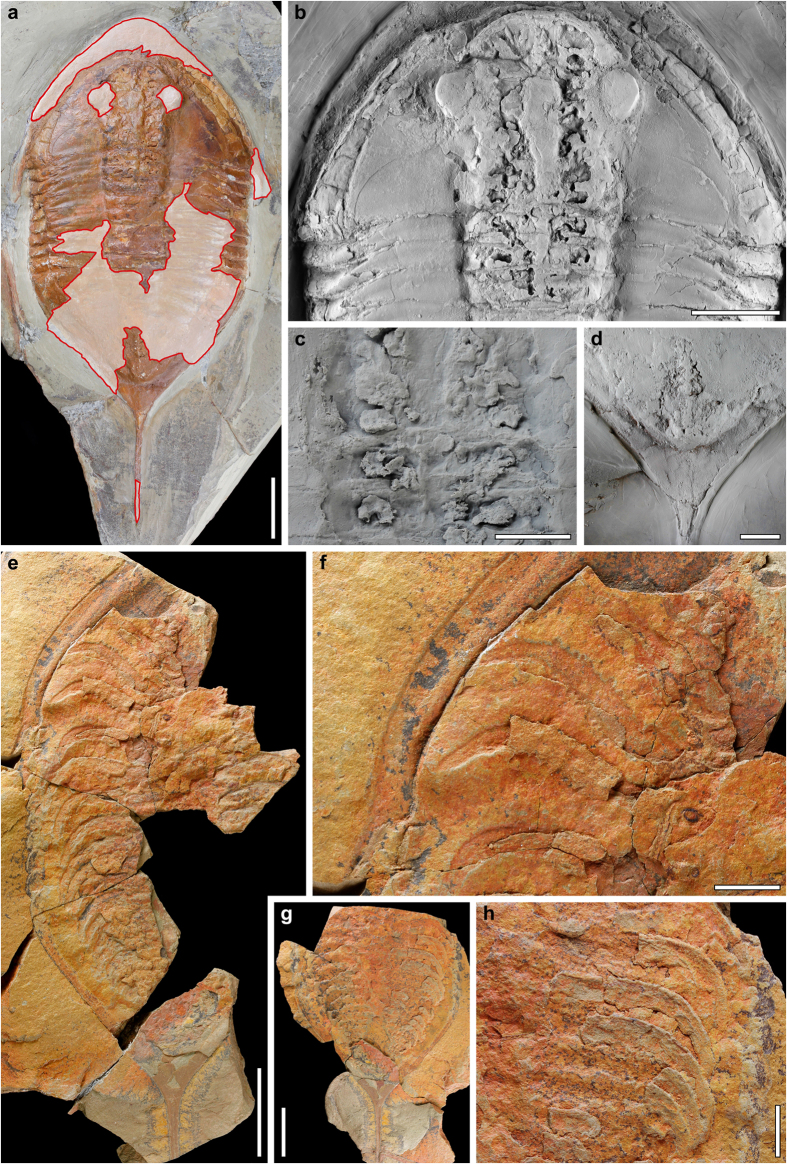
*Megistaspis (Ekeraspis) hammondi* with soft-body preservation from the late Tremadocian Fezouata Lagerstätte (Moroccan Anti-Atlas). (**a***–***d**) MGM-6755X, with digestive structures. (**a**) Overall view of specimen, with restored areas outlined in red and in lighter shade (for detailed indication of type of restoration see Supplementary Fig. 2); (**b**) detail of cephalic area and first thoracic segments with crop, intestine and digestive caeca; (**c**) latex cast of posterior cephalic and anterior thoracic caeca; (**d**) detail of the tip of the pygidial axis with remains of intestine and possible ?caeca. (**e**–**h**) MGM-6756X, part (**e**,**f**) and counterpart (**g**,**h**), of a specimen with preserved endopods. (**e**) Ventral right-hand side. (**f**) Detail of the cephalic and first thoracic endopods. (**g**) Counterpart of pygidium and five thoracic segments. (**h**) Detail of the last five thoracic endopods in positive relief. (**b–d**) have been whitened with MgO. Scale bar, 30 mm for (**a**,**e**,**g**); 20 mm for (**b**); 10 mm for (**c**,**d**,**f**,**h**).

The first specimen studied was the internal mould of a complete exoskeleton (Fig. 1a), partially reconstructed (Supplementary Fig. 2), which shows the internal anatomy in the glabellar–anterior thoracic axis and the posterior end of the pygydial axis. The digestive system can be recognized under the carapace of the glabellar area (Fig. 1b). The axial tube consists of an 8 mm-wide (tr.) crop (one-third of glabellar width), which extends from the anterior (exsagittal) edge of the eyes, tapering to 4 mm at the posterior end of the cephalon. This is followed by a 3 mm-wide intestine, preserved up to the third thoracic segment (Fig. 1b) and reappearing for 22 mm just before the axial end of the pygidium (Fig. 1d). Anterior to the crop, there seem to be two small, bilaterally-symmetrical branching caeca, which occupy the whole anterior region below the glabella. Behind the eyes, we can recognize at least 4 pairs of laterally-oriented, lobed caeca, whose tips are aligned longitudinally (posterior ones lengthen transversely as crop tapers). The bilateral position, together with the consistent size and similar shape of these structures suggests that these are true body features rather than irregular fractures of the exoskeleton revealing underlying cavities. The digestive caeca under the thoracic axis are also preserved in the first three segments (Fig. 1c), and are similar in size and shape to the posteriormost cephalic pair. The posterior end of the intestine seems to preserve three (?) pairs of small, simple caeca below the posterior part of the pygidial axis. However, due to the quality of preservation it cannot be ascertained whether caeca were present along the whole length of the digestive tract.

The crop and intestine present a positive dorsal relief, while the caeca have a negative ventral relief. The former have the same texture and grain size as the matrix, indicating active sediment ingestion or rapid sediment infill. The digestive caeca were presumably permineralized (possibly pyritized) in the early diagenetic stages, preserving their shape and relief. This has been described in other trilobites and soft-bodied arthropods and is attributed to these digestive structures being chemically enriched in life and prone to preferential mineralization^10^,^11^. These structures were later altered and eventually left the void observable now.

### Appendages

**Figure 2:**
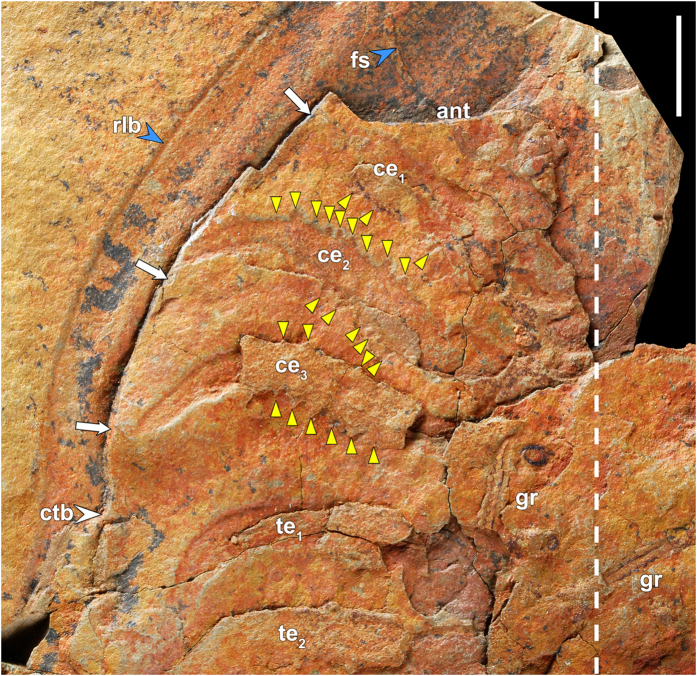
*Megistaspis (Ekeraspis) hammondi* with preserved cephalic and thoracic appendages from the late Tremadocian Fezouata Lagerstätte (Moroccan Anti-Atlas). Interpretation of specimen in Fig. 1f (MGM-6756X). View shows ventral structures in right-hand side: antenna (ant), three cephalic endopods (ce_1–3_) and first thoracic endopods (te_1–2_). Appendages are recognised by iron-stained external moulds, but some still preserve the casts (ce_1_, ce_3_, te_1_, te_2_). The second and third cephalic endopods are heavily armoured with dorsal and ventral spines (yellow arrowheads). Other recognisable features are facial suture (fs), the sediment infill (white arrows) under the right librigena (rlb) and the cranidium-thoracic boundary (ctb). The dashed line corresponds to the sagittal plane of the specimen, while ‘gr’ indicates fragments of graptolite stipes that were lying on the seafloor under the trilobite. Scale bar, 10 mm.

The second specimen of *M. (E.) hammondi* preserved a complete set of endopods, but no exopods (Fig. 1e–h). The specimen is incomplete, only preserving the right-hand side of the animal, from the base of the antennae to the last pygidial appendage. Its counterpart (Fig. 1g) is composed of the posterior 5 thoracic segments plus the pygidium. The endopods are preserved as external moulds of the dorsal side and some are also casts in positive relief (Fig. 1f,h). The endopods show some degree of heteropody: the cephalic ones are larger and heavier than the thoracic and pygidial ones and they are armed by dorsal and ventral spines on the proximal podomeres (Fig. 2). Podomere boundaries are not recognizable, but the spines probably correspond to podomeres 2–4, while the distal podomeres (5–6) appear smooth. The spines are stout and triangular; in the best-preserved cephalic appendage (2^nd^), a maximum of 11 spines can be counted dorsally and 7 ventrally (Figs 1f and 2). These spines are also recognizable in the cast of the third cephalic appendage. No spines are recognisable in the preserved thoracic and pygidial appendages. A total of 10 pygidial appendages can be recognized, which is more than the pygidial axial rings and pleural ribs (up to 7 and 5–6, respectively^8^). This is not a rare phenomenon in trilobites where appendages are known, like *Triarthrus*^12^. This decoupling between observed pygidial segments and number of pygidial appendages could be especially significant in those trilobites with effaced pygidial features like asaphoids and illaenoids.

**Figure 3:**
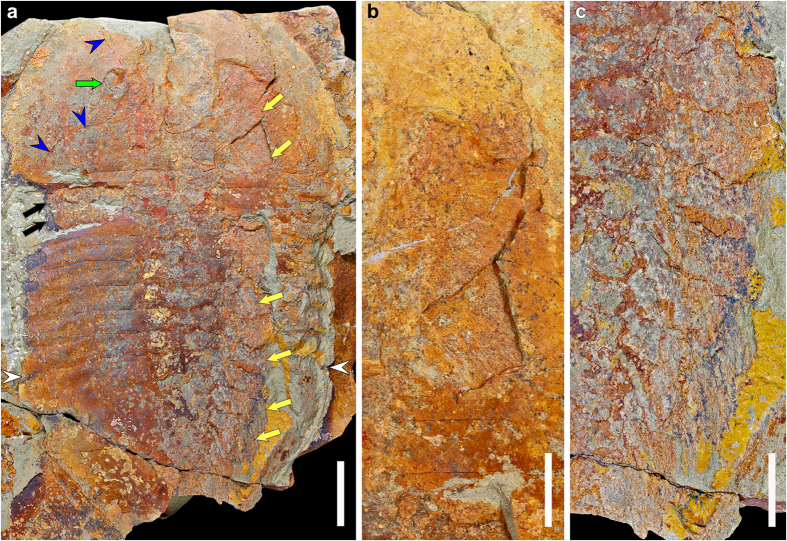
*Megistaspis (Ekeraspis) hammondi* with preserved endopods and exopods from the late Tremadocian Fezouata Lagerstätte (Moroccan Anti-Atlas). Specimen MGM-6757X (in dorsal aspect) preserving exopodal setae. (**a**) Overall view of specimen. Yellow arrows point to setae; black arrows indicate first and second thoracic endopods; green arrow points to left eye; blue arrowheads indicate the facial suture; white arrowheads indicate the thoracic-pygidial boundary. (**b**) Detail of the setae fringing the right cephalic exopods, exposed through a break in the cranidium. (**c**) Detail of the setae from right thoracic and pygidial exopods. The slab’s underside contains a well-preserved specimen of the graptolite “*Tetragraptus*” *bulmani* Thomas (=*T. longus* Lindhom), a very common species in the *Araneograptus murrayi* Biozone of the Fezouata Shale. Scale bar, 20 mm for (**a**); 10 mm for (**b**,**c**).

The third specimen displaying non-mineralized structures (Fig. 3) is an almost-complete, articulated carapace. Only the anterior portion of the cephalon and the posterior region of the pygidium are missing. The specimen is slightly flattened, and the dorsal cuticle has been lost in some regions, exposing the underlying soft-parts. The first and second left pleurae preserved their respective endopods with their tips folded underneath. Though most significantly, on the right side of the carapace, the cranidium, the thoracic pleurae and the pygidial pleural fields have split to reveal the postero-laterally oriented fine lamellae fringing the exopods (Fig. 3b). The lamellae of the posterior-most pygidial exopods, if complete in the specimen in Fig. 3a, were quite short, terminating before the border of the pygidium (Fig. 3c).

## Discussion

The digestive system of *M. (E.) hammondi* differs from the types described to date in trilobites^10^,^11^. It has lateral metameric-paired digestive caeca (recently^11^ referred to as “type 1”) of similar shape and relative size, but these extend to the posterior end of the midgut (pygidial axis), but it also has a well-defined crop (twice as wide as the intestine), characteristic of “type 2”, which lacks digestive caeca. This is the first instance of digestive caeca described among typical asaphids, where simple guts (“type 2”) have been recognized in the genera *Isotelus* and *Birmanites*, where the cephalic portion of the alimentary canal is not preserved^13^. However, these and other asaphid genera, where the presence of auxiliary muscular impressions in the glabella suggest the existence of a crop^11^, differ from *Megistaspis* in having a forked hypostome, related with predatory/scavenging habits^14^, although such an interpretation has not been undisputed^15^. Based on the gut preserved with the same texture as the matrix surrounding the fossil, the non-forked hypostome of *Megistaspis*^8^,^16^ and the slight heteropody in the cephalic appendages we can infer a detritus feeding habit for *M. (E.) hammondi*. This feeding habit is very common among other benthic trilobites^14^. A gut tract preserved under the pygidial axis has been also described in the asaphid *Isotelus*^17^, but there was no evidence of digestive caeca.

**Figure 4:**
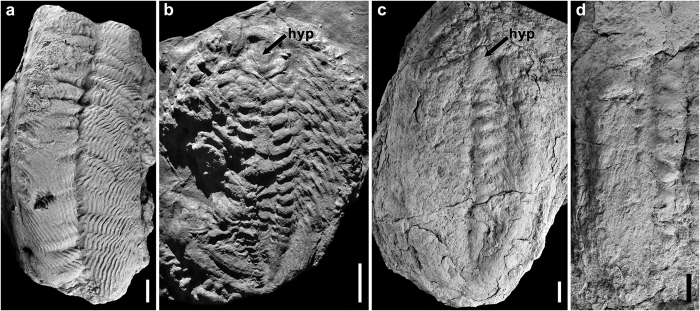
Behavioural traces attributed to certain Ordovician asaphid trilobites, reflecting ventral anatomy and preserved as convex hyporeliefs in sandstone. (**a**) Trail: *Cruziana rugosa* d’Orbigny, from early Dapingian of the Zenta Range near Laguna Verde, Argentina (range of the ichnospecies: Floian to Sandbian), MGM-6760X. (**b**) Resting burrow, *Rusophycus morgati* Baldwin, Armorican Sandstone (Floian) from the Armorican Massif, western France, interpreted as made by the trilobite *Ogyginus*^14^, IGR-114954. (**c,d**) Resting burrows*, Rusophycus carleyi* (James), upper Fezouata Shale (early Floian beds), from SW Ouzina, southern Tafilalt (eastern Anti-Atlas, Morocco), locally associated with the trilobite *Asaphellus*^23^. (**c**) MGM-6759X. (**d**) MGM-6758X, detail of traces attributed to endopodal spines along all the walking legs and impression of coxae to the right. hyp, hypostome impression; (**a**,**c**,**d**) have been whitened with MgO. Scale bar, 10 mm for (**a**,**c**,**d**); 20 mm for (**b**).

The cephalic appendages observed in *Megistaspis* are larger than the thoracic and pygidial ones, and are armed by dorsal and ventral spines in the proximal podomeres. The slight heteropody of the Moroccan *Megistaspis* may be related with a particular burrowing style, deduced for certain Ordovician trilobites from the trace fossil record. For example, *Cruziana rugosa* d’Orbigny displays combed dig-marks with up to twelve sharp-crested scratches, forming sets separated by transverse shelves (Fig. 4a). This has been interpreted as discontinuous ploughing of a trace-maker burrowing in head-down (procline) position, with multiple claws or spines on the tip of one or more of its endopodal podomeres^18^. In both surficial and endostratal variants of *C. rugosa*, the well-developed sets of scratches would questionably be the result of a strongly serrated tip of the pretarsus^18^, but more likely be made by the heavier-built, proximal podomeres of the cephalic appendages, especially when these where spine-bearing as in *Megistaspis*. The sets of parallel scratches make little sense as the “fingerprints” of individual distal claws. The production of this trace has been attributed^19^ to trilobites with anterior appendages greater in size that the posterior ones, and equipped with multiple or serrated claws. However, other authors^20^ have interpreted *C. rugosa* as the digging action of a single pair of broad, multidigited anterior appendages, producing dig marks that remain separate, and for the most part undisturbed by successive excavation strokes. However, in the absence of any record of a trilobite equipped with these structures (see ref. 21 for extensive discussion), this ichnospecies of *Cruziana* is indeed consistent with the spines observed in the podomeres of the cephalic appendages of *M. (E.) hammondi* (Figs 1f and 2), which could have produced sets of parallel striae or “combs” (Fig. 4a). The stout spines of this trilobite species are more likely associated with a digging activity than the sharp, fine spines of trilobites such as *Olenoides*, which probably had a grasping and ripping function for predation or scavenging^22^. Although those fine spines are present in some deep-water Ordovician olenid trilobites such as *Triarthrus*^12^,^23^, in the asaphid trilobite studied here, the direct evidence does not suggest this as the principal feeding habit. Furthermore, *Cruziana rugosa* is regarded as a combined feeding and locomotion trace (‘pascichnion’) most likely produced by the food searching strategy of asaphoid trilobites^18^,^24^. This attribution is based in the large size reached by the traces (up to 26 cm wide^21^), only compatible with the size of some of these trilobites distributed around mid- to high-latitude Gondwanan regions, and also due to the local co-occurrence in the same levels of *C. rugosa* and the trilobite *Ogyginus*^25^. However, among asaphoid trilobites there are two types of hypostomes: those with forked hypostomes, possibly related with predatory or scavenging habits on worms and other small invertebrates^14^, and those with normal hypostomes (such as *Ekeraspis, Asaphellus* and *Ogyginus*), which were probably deposit feeders with microphagous habits (“mudtrophobacterivory” feeding^21^), being the most likely candidates as trace-makers of *C. rugosa*.

Some ovate resting traces of *Rusophycus*, display the impression of most of the ventral morphology of their supposed asaphoid producer. The fine, backward-oriented, oblique fine crests of *Rusophycus morgati* and *R. carleyi* (Fig. 4b–d), have been attributed to endopodal spines on possibly all the endopods^26^. These Lower Ordovician trilobite traces recorded in the high-latitude peri-Gondwanan shelves, had previously been assigned to predatory forms^14^, but have since been reinterpreted as moulting burrows^18^,^27^. The trace-makers lack forked hypostomes and have been assigned to *Ogyginus*^14^ and *Asaphellus*^26^. The ventral soft-body morphology described herein may have produced similar resting traces, except for *Megistaspis*’ long caudal spine, which could have been directed slightly upward in life.

## Conclusions

Some early Palaeozoic trilobites^10^,^11^ and numerous non-mineralized arthropods^28^ had digestive structures that were prone to early permineralization, possibly due to being enzymatically rich areas^11^,^25^. Sometimes it was the digestive tract that was preserved, while others it was the glands connected to it, mainly the mid-gut glands. These mid-gut glands are paired, metameric structures located on the sides of the intestine, and in modern arthropods occur in groups with predatory habits, such as chelicerates and remipedes^25^, but its combination with a crop (for storage of food), non-forked hypostome and stout appendage spines suggests a mixed feeding habit for *Megistaspis*. This original combination of crop and digestive caeca might be regarded as a new, “type 3” trilobite digestive system^11^. These Moroccan fossils constitute the first recognition of heteropody in trilobite walking appendages, where *Megistaspis* would primarily use its spiny cephalic appendages in digging for food, with a comb-like movement already inferred for the producers of the *Cruziana rugosa* group of traces, an ichnogroup widely distributed throughout the Ordovician of Gondwana.

## Methods

Five of the six examined specimens are housed in the Museo Geominero of Madrid, Spain (prefixed MGM), and one at the Geological Museum of the University of Rennes, France (prefixed IGR). All photographs were taken with a Canon EOS7D digital SLR camera equipped with a Canon MP-E 65 mm 1–5x macro lens. Some of the specimens were whitened with MgO before photographing (Figs 1b–d and 4a,c,d), and digestive structures of specimen MGM-6755X were replicated as latex casts and whitened (Fig. 1c). Illustrations composed and processed, to homogenise light intensity across figures, with Adobe Photoshop CS3. Supplementary Fig. 1 was drafted with CorelDRAW 12 and Adobe Photoshop CS3.

## Additional Information

**How to cite this article**: Gutiérrez-Marco, J. C. *et al*. Digestive and appendicular soft-parts, with behavioural implications, in a large Ordovician trilobite from the Fezouata Lagerstätte, Morocco. *Sci. Rep.* **7**, 39728; doi: 10.1038/srep39728 (2017).

**Publisher's note:** Springer Nature remains neutral with regard to jurisdictional claims in published maps and institutional affiliations.

## Supplementary Material

Supplementary Figures

## References

[b1] Van Roy P. . Ordovician faunas of Burgess Shale type. Nature 465, 215–218 (2010). 20463737 10.1038/nature09038

[b2] Van Roy P., Briggs D. E. G. & Gaines R. R. The Fezouata fossils of Morocco; an extraordinary record of marine life in the Early Ordovician. J. Geol. Soc. 172, 541–549 (2015).

[b3] Martin E. . The Lower Ordovician Fezouata Konservat-Lagerstätte from Morocco: age, environment and evolutionary perspectives. Gondwana Res. 34, 274–283 (2016).

[b4] Van Roy P., Daley A. C. & Briggs D. E. G. Anomalocaridid trunk limb homology revealed by a giant Ordovician filter-feeder with paired lateral flaps. Nature 522, 77–80 (2015). 25762145 10.1038/nature14256

[b5] Vinther J., Van Roy P. & Briggs D. E. G. Machaeridians are Palaeozoic armoured annelids. Nature 451, 185–188 (2008). 18185586 10.1038/nature06474

[b6] Lefebvre B. & Botting J. P. First report of the mitrate *Peltocystis cornuta* Thoral (Echinodermata, Stylophora) in the Lower Ordovician of central Anti-Atlas (Morocco). Ann. Paléont. 93, 183–198 (2007).

[b7] Corbacho J. & Vela J. A. Giant trilobites from Lower Ordovician of Morocco. Batalleria 15, 3–32 (2010).

[b8] Vidal M. Trilobites (Asaphidae et Raphiphoridae) de l’Ordovicien inférieur de l’Anti-Atlas, Maroc. Palaeontographica A 251, 39–77 (1998).

[b9] Martin E. L. O. . Biostratigraphic and palaeonvironmental controls on the trilobite associations from the Lower Ordovician Fezouata Shale of the Central Anti-Atlas, Morocco. Palaeogeogr., Palaeoclimat., Palaeoecol 460, 142–154 (2016).

[b10] Chatterton B. D. E., Johanson Z. & Sutherland G. Form of the trilobite digestive system: alimentary structures in *Pterocephalia*. J. Paleont. 68, 294–305 (1994).

[b11] Lerosey-Aubril R. . Controls on gut phosphatisation: the trilobites from the Weeks Formation Lagerstätte (Cambrian; Utah). PLoS ONE 7, 1–9 (2012). 10.1371/journal.pone.0032934 PMC3303877 22431989

[b12] Whittington H. B. & Almond J. B. Appendages and habits of the Upper Ordovician trilobite *Triarthrus eatoni*. Phil. Trans. R. Soc. London B 317, 1–46 (1987).

[b13] Fatka O., Lerosey-Aubril R. & Rak Š. Fossilised guts in trilobites from the Upper Ordovician Letná Formation (Prague Basin, Czech Republic). Bull. Geosci. 88, 95–104 (2013).

[b14] Fortey R. A. & Owens R. M. Feeding habits in trilobites. Palaeontology 42, 429–465 (1999).

[b15] Hegna T. A. The function of forks: *Isotelus*-type hypostomes and trilobite feeding. Lethaia 43, 411–419 (2010).

[b16] Jaanusson V. Untersuchungen über baltoskandische Asaphiden. III. Über die Gattungen *Megistaspis* n. nom. und *Homalopyge* n. gen. Bull. Geol. Inst. Uppsala 36, 59–77 (1956).

[b17] English A. M. & Babcock L. E. Feeding behaviour of two Ordovician trilobites inferred from trace fossils and non-biomineralised anatomy, Ohio and Kentucky, USA. Mem. Ass. Austral. Palaeontol. 34, 537–544 (2007)

[b18] Seilacher A. Trace fossil analysis (Springer-Verlag, 2007).

[b19] Seilacher A. *Cruziana* stratigraphy of “non fossiliferous” Palaeozoic sandstones. In Crimes T. P. & Harper J. C. (eds). Trace fossils, Geol. J. Spec. Iss. 3, 447–476 (1970).

[b20] Baldwin C. T. The stratigraphy and facies associations of trace fossils in some Cambrian and Ordovician rocks of north western Spain. In Crimes T. P. & Harper J. C. (eds). Trace fossils 2, Geol. J. Spec. Iss. 9, 9–40 (1977).

[b21] Whittington H. B. Exoskeleton, moult stage, appendage morphology, and habits of the Middle Cambrian trilobite *Olenoides serratus*. Palaeontology 23, 171–204 (1980).

[b22] Neto de Carvalho C. Roller coaster behavior in the *Cruziana rugosa* group from Penha Garcia (Portugal): Implications for the feeding program of trilobites. Ichnos 13, 255–265 (2006).

[b23] Boyer D. L. & Mitchell C. E. Aligned trace fossils from the Utica Shale: implications for mode of life and feeding in the trilobite *Triarthrus beckii*. Lethaia., doi: 10.1111/let.12177 (2016).

[b24] Bergström J. Lower Paleozoic trace fossils from eastern Newfoundland. Canadian Journal of Earth Sciences 13, 1613–1633 (1976).

[b25] Mángano M. G. & Waisfeld B. Looking for the usual suspects: Trilobites as *Cruziana-Rhusophycus* producers in lower Paleozoic sandstones of northwest Argentina. Abstracts First International Congress on Ichnology (Ichnia 2004), Trelew (Argentina), 50–51 (2004).

[b26] Gibb S., Chatterton B. D. E. & Gingras M. K. *Rusophycus carleyi* (James, 1885), trace fossils from the Lower Ordovician of southern Morocco, and the trilobites that made them. Ichnos 17, 271–283 (2010).

[b27] Seilacher A., Gibb S. & Hughes N. Trilobite trace fossils made for moulting? Palaeontological Society of India 60, 27–32 (2015).

[b28] Butterfield N. J. *Leanchoilia* guts and the interpretation of three-dimentional structures in Burgess Shale-type fossils. Paleobiology 28, 155–171 (2002).

[b29] Vannier J. & Chen J.-Y. Digestive system and feeding mode in Cambrian naraoiid arthropods. Lethaia 35, 107–120 (2002).

[b30] Vannier J. . Sophisticated digestive systems in early arthropods. Nat. Commun. 5, 3641 (2014). 24785191 10.1038/ncomms4641

