# Supplementary material for: Digestive and appendicular soft-parts, with behavioural implications, in a large Ordovician trilobite from the Fezouata Lagerstätte, Morocco
Source: Sci Rep. 2017 Jan 10;7:39728. doi: 10.1038/srep39728 (PMC5223178; doi:10.1038/srep39728)
Supplement: Supplementary Figures [file srep39728-s1.pdf]

Digestive and appendicular soft-parts, with behavioural implications, in a large Ordovician trilobite from the Fezouata Lagerstätte, Morocco

Juan C. Gutiérrez-Marco, Diego C. García-Bellido, Isabel Rábano & Artur A. Sá

SUPPLEMENTARY FIGURES 1–3

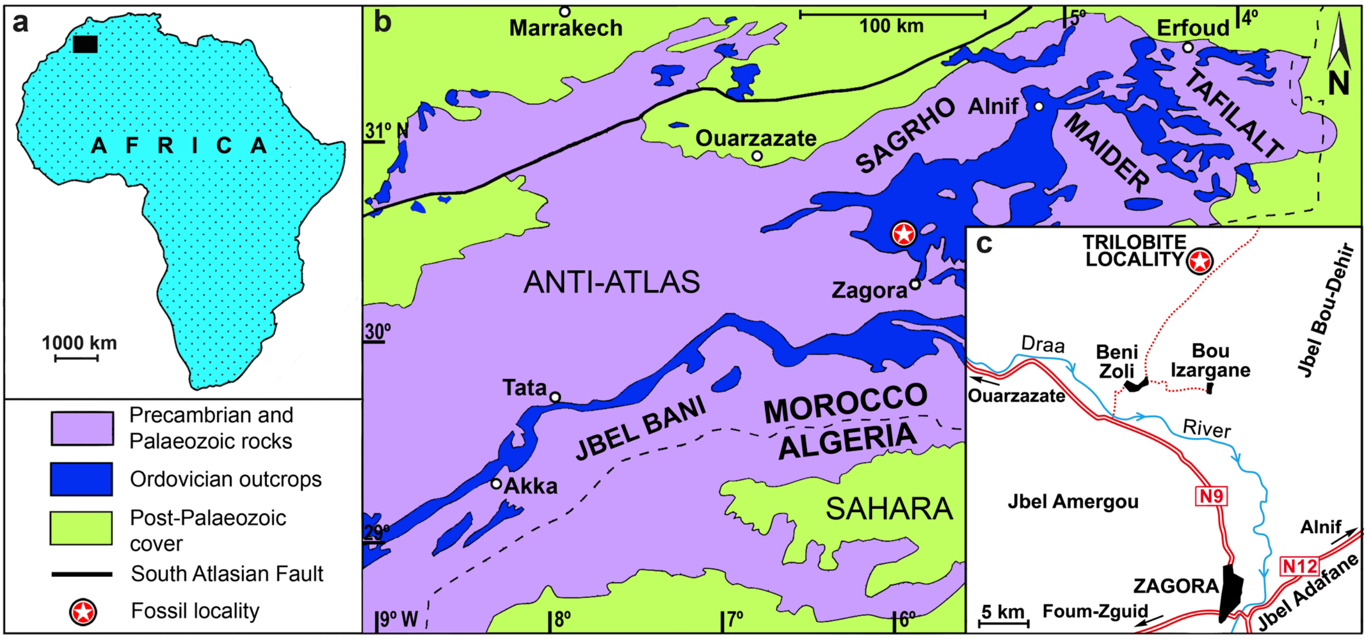

Supplementary Figure 1. Fossil locality. (**a**) Map indicating the position of the studied region in Africa. (**b**) Geological sketch map of the central and eastern Anti-Atlas of Morocco, with indication of Ordovician outcrops and the position of the fossil locality (star). (**c**) Sketch map of the Zagora area including the trilobite site (star). [**a** and **b** partially modified from Rábano, I., Gutiérrez-Marco, J. C. & García-Bellido, D. C. A remarkable illaenid trilobite from the Middle Ordovician of Morocco. *Bulletin of Geosciences* **89**, 365–374 (2014)]. This figure has been drafted with Corel Draw 12 (www.corel.com) and Adobe Photoshop CS3 (www.adobe.com/CreativeCloud/Photoshop).

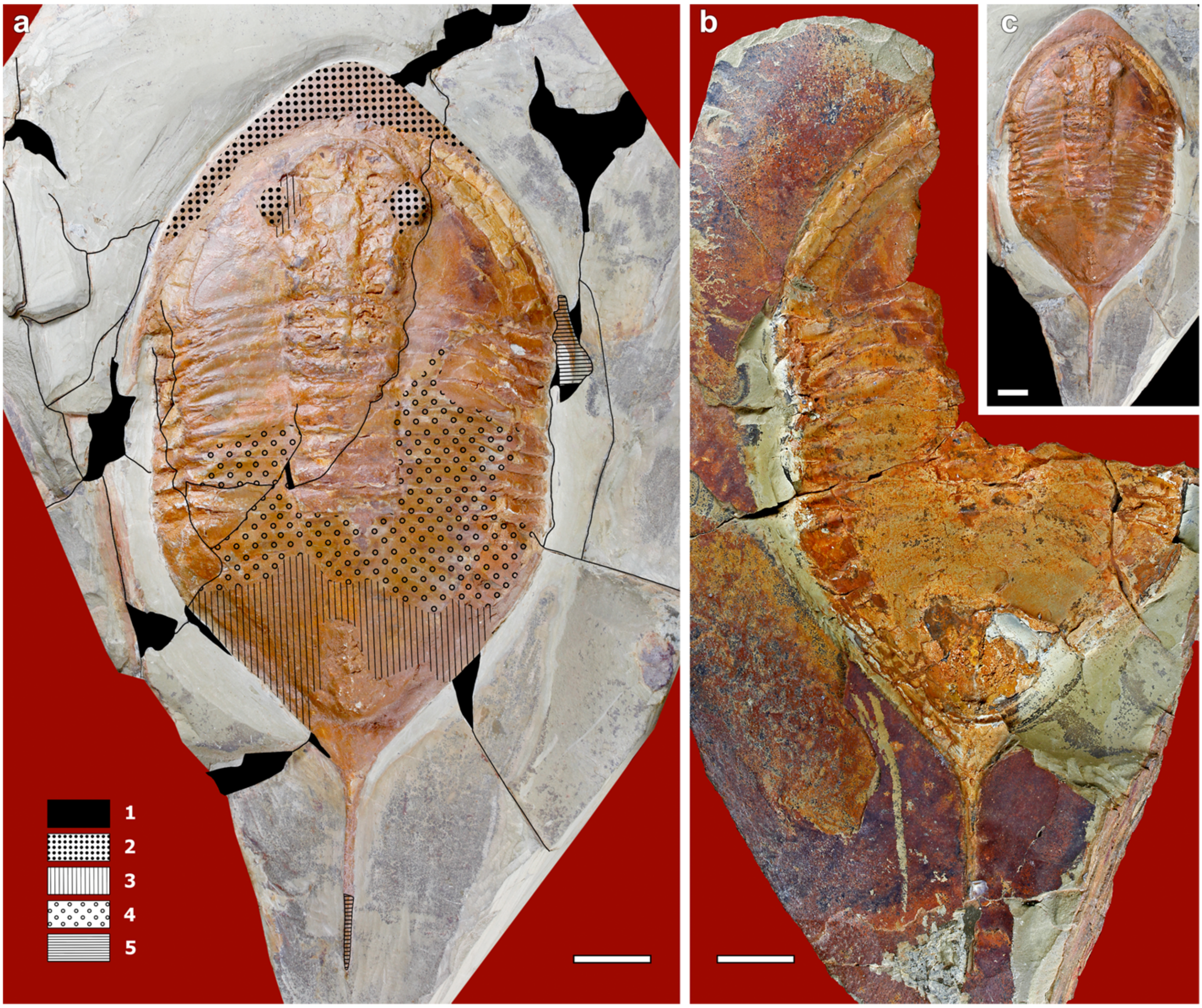

Supplementary Figure 2. ***Megistaspis*** **(*Ekeraspis*)** ***hammondi*** **from the late Tremadocian Fezouata Lagerstätte (Moroccan Anti-Atlas).** (**a**) Convex part of MGM-6755X (reproduced also on Fig. 1a) with indication of the intact parts, as well as those glued and reconstructed by the fossil dealers. (**b**) Incomplete external mould (concave counterpart) of articulated specimen MGM-6755X. (**c**) Overview of complete, reconstructed specimen MGM-6755X. Legend: 1, lost parts filled with mix of rock dust and epoxy resin; 2, extensively reconstructed parts, made with coloured epoxy resin; 3, slightly reconstructed parts; 4, polished surfaces of the internal mould; 5, pieces transferred from the external mould. Scale bar, 20 mm.

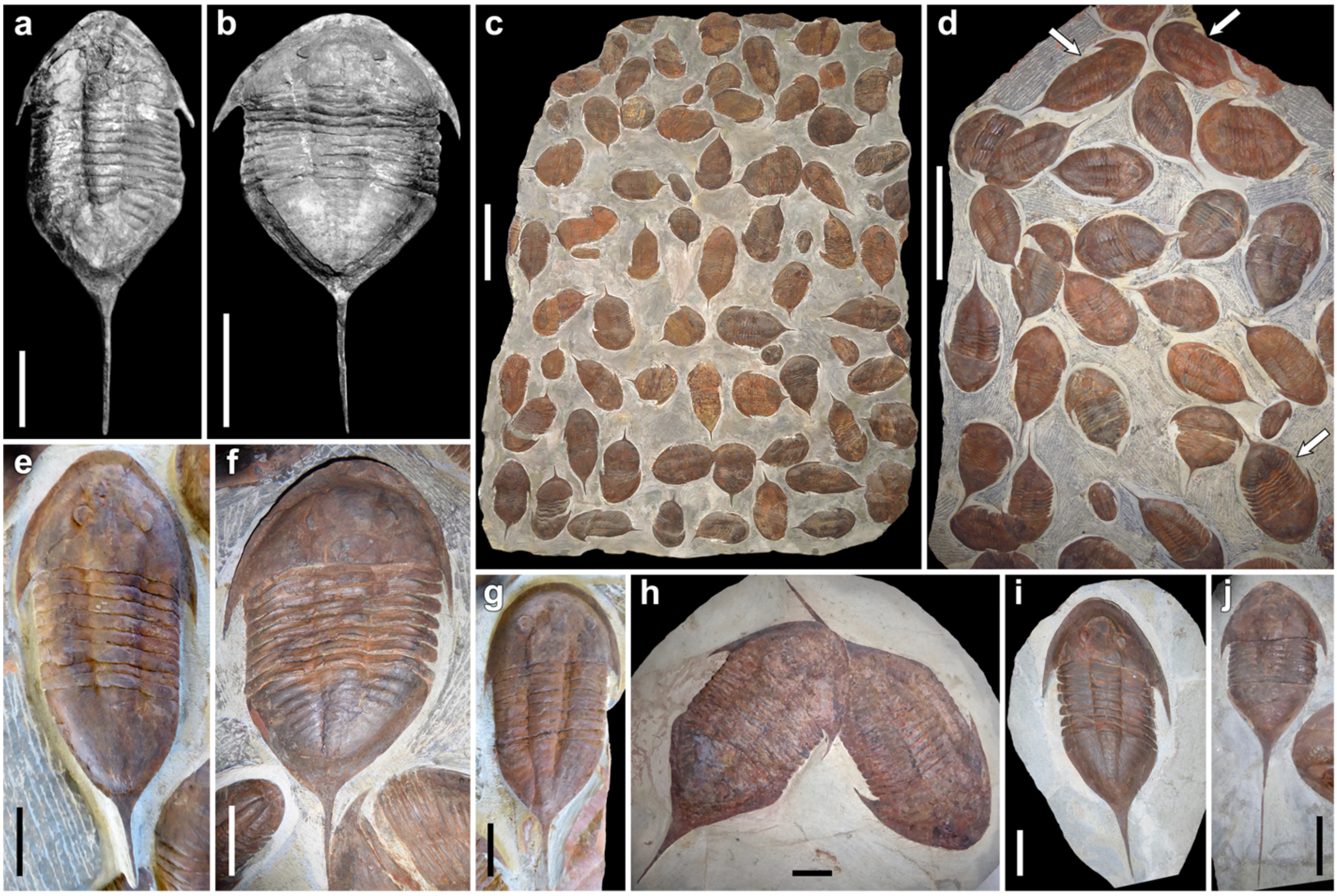

Supplementary Figure 3. Important samples of *Megistaspis* (*Ekeraspis*) *hammondi* from the Lower Ordovician of the Moroccan Anti-Atlas. These specimens show morphological variations due to crude reconstructions and/or diverse compactional deformations. (**a**) Holotype specimen of *M*. (*E*.) *h*. *hammondi* (NHM It28344, reproduced from Corbacho & Vela 2010[7], pl. 4A), with damaged glabella and pygidial rachis. (**b**) Holotype of *M*. (*E*.) *h*. *forteyi* (NHM It28343, from Corbacho & Vela 2010[7], pl. 5A), with bent thorax and downward-directed cephalon. (**c**,**d**) Two large slabs with aggregation of specimens imitating bedding plane assemblages, for sale in October 2015 at the Kasmi-Achour shop (**c**) and Tahiri Museum (**d**) of Erfoud (Morocco). Arrows in **d** indicate specimens figured in **e**, **f** and **g**. (**e**–**g**) Detail of diverse specimens from the large slab in **d**. (**h**) Two rather well-preserved specimens belonging to the permanent exhibition on the Tahiri Museum of Erfoud. (**i**–**j**) Complete specimens on sale at the Kasmi-Achour fossil shop, showing exagerated reconstructions of genal and caudal spines, respectively. **a**,**b** are housed in the palaeontological collections of the Natural History Museum, London. Scale bar, 50 mm for **a**,**b**,**e**–**j**; 250 mm for **c**,**d**.
